# Effectiveness of antipsychotics for managing agitated delirium in patients with advanced cancer: a secondary analysis of a multicenter prospective observational study in Japan (Phase-R)

**DOI:** 10.1007/s00520-024-08352-2

**Published:** 2024-02-08

**Authors:** Ken Kurisu, Shuji Inada, Isseki Maeda, Hiroyuki Nobata, Asao Ogawa, Satoru Iwase, Megumi Uchida, Tatsuo Akechi, Koji Amano, Nobuhisa Nakajima, Tatsuya Morita, Masahiko Sumitani, Kazuhiro Yoshiuchi

**Affiliations:** 1 Department of Stress Sciences and Psychosomatic Medicine, Graduate School of Medicine, The University of Tokyo, Tokyo, Japan; 2 Department of Psychosomatic Medicine, Saitama Cancer Center, Saitama, Japan; 3 Department of Palliative Care, Senri-Chuo Hospital, Osaka, Japan; 4 Arata Clinic, Nagasaki, Japan; 5 Department of Psycho-Oncology Service, National Cancer Center Hospital East, Kashiwa, Chiba Japan; 6 Department of Palliative Medicine, Saitama Medical University, Iruma, Saitama Japan; 7 Department of Psychiatry and Cognitive-Behavioral Medicine, Graduate School of Medical Sciences, Nagoya City University, Nagoya, Aichi Japan; 8 Center for Psycho-Oncology and Palliative Care, Nagoya City University Hospital, Nagoya, Aichi Japan; 9 Palliative and Supportive Care Center, Osaka University Hospital, Osaka, Japan; 10 Division of Community Medicine and International Medicine, University of the Ryukyus Hospital, Okinawa, Japan; 11 Department of Palliative and Supportive Care, Seirei Mikatahara General Hospital, Hamamatsu, Shizuoka Japan; 12 Research Association for Community Health, Hamamatsu, Shizuoka Japan; 13 Department of Pain and Palliative Medicine, The University of Tokyo Hospital, Tokyo, Japan

**Keywords:** Delirium, Agitation, Richmond agitation-sedation scale, Olanzapine, Pharmacotherapy, Advanced cancer

## Abstract

**Purpose:**

Delirium is a common and serious comorbidity in patients with advanced cancer, necessitating effective management. Nonetheless, effective drugs for managing agitated delirium in patients with advanced cancer remain unclear in real-world settings. Thus, the present study aimed to explore an effective pharmacotherapy for this condition.

**Methods:**

We conducted a secondary analysis of a multicenter prospective observational study in Japan. The analysis included patients with advanced cancer who presented with agitated delirium and received pharmacotherapy. Agitation was defined as a score of the Richmond Agitation-Sedation Scale for palliative care (RASS-PAL) of ≥ 1. The outcome was defined as -2 ≤ RASS-PAL ≤ 0 at 72 h after the initiation of pharmacotherapy. Multiple propensity scores were quantified using a multinomial logistic regression model, and adjusted odds ratios (ORs) were calculated for haloperidol, chlorpromazine, olanzapine, quetiapine, and risperidone.

**Results:**

The analysis included 271 patients with agitated delirium, and 87 (32%) showed -2 ≤ RASS-PAL ≤ 0 on day 3. The propensity score-adjusted OR of olanzapine was statistically significant (OR, 2.91; 95% confidence interval, 1.12 to 7.80; *P* = 0.030).

**Conclusions:**

The findings suggest that olanzapine may effectively improve delirium agitation in patients with advanced cancer.

**Supplementary Information:**

The online version contains supplementary material available at 10.1007/s00520-024-08352-2.

## Introduction

Delirium is a prevalent and serious comorbidity in patients with advanced cancer attributable to several factors, such as coexisting physical abnormalities and opioid use [1]. Small doses and short-term administration of antipsychotics are considered for reducing delirium symptoms when they cause severe distress or harm to others [1–3]. However, their effectiveness remains controversial [4–6].

In this context, we conducted a multicenter prospective observational study in Japan, called the Japan Pharmacological Audit Study of Safety and Effectiveness in Real-world (Phase-R), to investigate the effectiveness of pharmacotherapy for delirium in real-world settings [7]. Using the Delirium Rating Scale-Revised-98 (DRS-R98) as the primary outcome, we found an association between quetiapine usage and DRS-R98 score improvement [7]. In contrast, a machine learning model trained with the same dataset revealed that the selection of drugs had no influence on predicting improvement in the DRS-R98 score, whereas the baseline delirium severity and specific precipitating factors were influential predictors [8].

The DRS-R98, which comprehensively assesses symptoms, including agitation and cognitive dysfunction [9], has been primarily employed in delirium research [10]. However, among those symptoms, agitation is notably distressing for patients, caregivers, and medical staff, necessitating effective management [11–14]. To examine the effective management of agitation, several studies have used the Richmond Agitation-Sedation Scale (RASS) as the primary outcome, a measure solely focused on agitation, instead of the DRS-R98 [15, 16]. Nonetheless, effective pharmacological management of agitated delirium in patients with advanced cancer in real-world settings remains inadequately explored.

Based on these findings, we conducted a secondary analysis of Phase-R data with the RASS as the study outcome, aiming to explore an effective pharmacological intervention for agitated delirium in patients with advanced cancer in real-world settings.

## Methods

### Phase-R database

The present study was a secondary analysis of Phase-R, a multicenter prospective observational study conducted at 14 palliative care units certified by the Japanese Society for Hospice and Palliative Care and 9 psycho-oncology settings within tertiary cancer hospitals or university hospitals in Japan between September 2015 and May 2016. Psycho-oncology settings were defined as those staffed with psychiatrists or psychosomatic physicians who provide consultations and liaisons for patients with cancer.

The Phase-R project included patients with advanced cancer who were diagnosed with delirium by a trained palliative care physician or psycho-oncologist based on the Diagnostic and Statistical Manual of Mental Disorders, 5th edition [17], and who received antipsychotics (chlorpromazine, haloperidol, olanzapine, perospirone, quetiapine, and risperidone), or trazodone, which is frequently prescribed for delirium in Japan [18]. Patients with postoperative delirium and those with alcohol or drug withdrawal delirium were excluded.

In this secondary analysis, we extracted patients who exhibited agitated delirium, as per the definition described below, from the Phase-R database.

The study protocol was approved by the Institutional Review Boards of Osaka University (approval number: 13295) and each participating institution. The requirement for informed consent was waived because the study collected data from records of usual clinical practice.

### Definition of agitated delirium and study outcome

In the Phase-R project, the RASS for Palliative Care (RASS-PAL), which is designed to evaluate the level of sedation and agitation in palliative care settings [19], was used. It includes scores ranging from -5 to + 4 on a 10-point scale, with each score indicating a level of sedation or agitation: -5 = unarousable, -4 = deep sedation, -3 = moderate sedation, -2 = light sedation, -1 = drowsy, 0 = alert and calm, + 1 = restless, + 2 = agitated, + 3 = very agitated, and + 4 = combative. The assessment using this scale was performed before (baseline) and 72 h after (day 3) the initiation of pharmacotherapy.

We defined agitation as a baseline RASS-PAL point of ≥ 1 and included patients who met the criteria in the analysis. Previous studies have used the degree of reduction in RASS points as the primary endpoint [15, 16] and -2 ≤ RASS ≤ 0 as the secondary endpoint [16]; however, excessively low RASS values indicate an uncommunicative state, which is undesirable for patients and caregivers [20, 21]. Consequently, using the value on day 3, we set -2 ≤ RASS-PAL ≤ 0 as the study outcome.

### Statistical analyses

We used a multinomial logistic regression model that was trained to predict the use of each drug to quantify multiple propensity scores for adjusting for potential confounders [22]. A practical guide was followed to select appropriate confounding variables [23], leading us to include variables associated with both outcome and treatment and those solely associated with the outcome while excluding those linked only to treatment but not to the outcome.

Ultimately, we included age, sex, ECOG Performance Status, baseline RASS-PAL score, setting (palliative care or psycho-oncology), physician-estimated prognosis, oral intake availability, and other risk factors categorized by Lipowski [24]. Potential direct factors were selected by palliative care physicians or psycho-oncologists, with multiple choices allowed, from the following options: opioids, drugs other than opioids, dehydration, non-respiratory infection, respiratory infection, organic damage to the central nervous system, hypoxia, liver failure, renal failure, electrolytes disturbance, and others. Regarding preparatory factors, relevant comorbidities, including brain tumor or metastasis, cerebrovascular diseases, and dementia, were included. As the facilitating factors, symptoms of fecal impaction, urinary retention, and average pain in the previous week measured by the Support Team Assessment Schedule were included.

Using the multiple propensity scores as a covariate, we developed a logistic regression model to quantify the adjusted odds ratio (OR) for each drug type. The classification of the drug type was determined after checking the sample size of the participants receiving each drug. Owing to the limited availability of non-oral antipsychotic drugs in Japan [25], we conducted a subgroup analysis stratified by oral intake availability.

All statistical analyses were performed using R version 4.3.1 (R Foundation for Statistical Computing, Vienna, Austria, 2023). The multinomial logistic regression model for multiple propensity scores was implemented using the “nnet” package (version 7.3–19). Statistical significance was set at *P* < 0.05.

## Results

### Descriptive statistics

In the Phase-R study, 271 patients presented with agitated delirium, defined as a baseline RASS-PAL point of ≥ 1. Among them, 87 (32%) showed -2 ≤ RASS-PAL ≤ 0 on day 3. Table 1 presents the descriptive statistics of the patients. Among the estimated direct factors, opioids were the most frequently reported (39.1%). Table 2 shows the number of participants receiving each drug and the dosage on day 3.

**Table 1:** Participants’ characteristics

	*N* = 271
Age, Mean (SD)	71.9 (10.7)
Male sex, *n* (%)	177 (65.3)
Setting, *n* (%)	
Psycho-Oncology Palliative Care	77 (28.4) 194 (71.6)
Primary tumor sites, *n* (%)	
Lung Esophagus/Stomach Liver/binary system/pancreas Colon/rectum Kidney/urinary system/prostate Breast Uterine/ovary Blood Others	68 (25.1) 36 (13.3) 49 (18.1) 31 (11.4) 23 (8.5) 11 (4.1) 16 (5.9) 13 (4.8) 24 (8.9)
Performance status, Mean (SD)	3.2 (0.8)
Estimated prognosis, *n* (%)	
Days Weeks Months	41 (15.1) 151 (55.7) 79 (29.2)
Comorbidities, *n* (%)	
Brain tumor or metastasis Cerebrovascular diseases Dementia	40 (14.8) 21 (7.7) 20 (7.4)
Direct factors, *n* (%)	
Opioids Drugs other than opioids Dehydration Non-respiratory infection Respiratory infection Organic damage to the central nervous system Hypoxia Liver failure Renal failure Electrolytes disturbance Others	106 (39.1) 42 (15.5) 23 (8.5) 41 (15.1) 30 (11.1) 31 (11.4) 63 (23.2) 49 (18.1) 23 (8.5) 25 (9.2) 20 (7.4)
Symptoms, *n* (%)	
Fecal impaction Urinary retention	21 (7.7) 7 (2.6)
STAS average pain, Mean (SD)	1.2 (1.0)
Oral intake available, *n* (%)	165 (60.9)
DRS-R98 severity score, Mean (SD)	
Baseline Day 3	18.4 (7.1) 17.4 (8.6)
RASS-PAL, Mean (SD)	
Baseline Day 3	1.49 (0.76) -0.54 (2.25)

*SD* standard deviation, *STAS* Support Team Assessment Schedule

**Table 2:** Pharmacotherapy used for management of delirium (*N* = 271)

	*N* (%)	Dosage on day 3 (mg/day), mean (SD)
Chlorpromazine	30 (11.1)	14.7 (12.2)
Haloperidol	107 (39.5)	7.4 (15.8)
Olanzapine	29 (10.7)	6.3 (5.3)
Perospirone	7 (2.6)	3.9 (2.0)
Quetiapine	45 (16.6)	60.1 (63.5)
Risperidone	49 (18.1)	1.3 (0.7)
Trazodone	4 (1.5)	37.5 (14.4)

Supplementary Fig. 1 illustrates the distribution of facilities according to the number of patients receiving each drug, indicating that olanzapine was predominantly administered in specific centers.

Due to the small sample size of patients receiving perospirone (*N* = 7) and trazodone (*N* = 4), these patients were excluded from the main analysis, resulting in a sample size of *N* = 260.

Furthermore, we conducted an additional analysis using three categories of antipsychotics: multi-acting receptor-targeted antipsychotics (MARTA; olanzapine and quetiapine), serotonin dopamine antagonists (SDA; perospirone and risperidone), and typical antipsychotics (chlorpromazine and haloperidol). In this supportive analysis, perospirone was included as an SDA (*N* = 7), leading to a total sample size of *N* = 267.

### Effectiveness of antipsychotics for the RASS-PAL point improvement

Table 3 shows the propensity score-adjusted OR for each drug. The adjusted OR for olanzapine was statistically significant (OR, 2.91; 95% confidence interval, 1.12 to 7.80; P = 0.030).

**Table 3:** Propensity score-adjusted odds ratios of each drug (*N* = 260)

	Odds ratio (95% CI)	P value
Haloperidol (*N* = 107)	1.00 (reference)	
Chlorpromazine (*N* = 30)	0.66 (0.20 to 1.98)	0.47
Olanzapine (*N* = 29)	2.91 (1.12 to 7.80)	**0.030**
Quetiapine (*N* = 45)	0.63 (0.21 to 1.84)	0.40
Risperidone (*N* = 49)	0.86 (0.33 to 2.26)	0.76

*CI* confidence interval

**Fig. 1:**
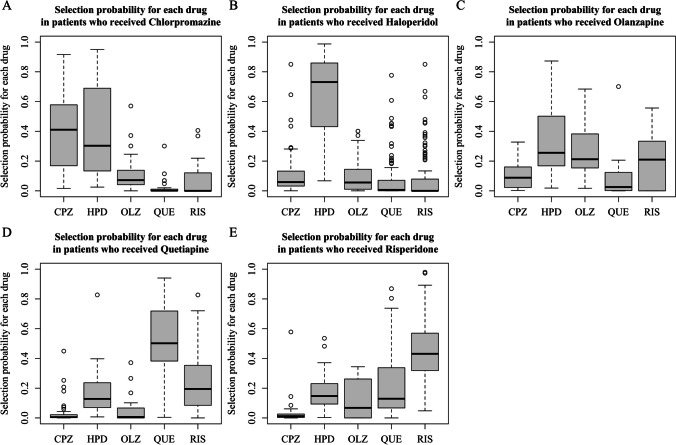
Box plots showing the distribution of multiple propensity scores (the probability of selecting each drug at the initiation of treatment) among patients receiving (**A**) Chlorpromazine, (**B**) Haloperidol, (**C**) Olanzapine, (**D**) Quetiapine, and (**E**) Risperidone

Figure 1 presents box plots for evaluating the overlap of multiple propensity score distributions. The distribution of scores in patients receiving haloperidol exhibited small overlaps, suggesting that confounding factors strongly influenced the selection of haloperidol [22].

Table 4 shows the adjusted ORs of the drugs classified as typical antipsychotics, MARTA, and SDA. The adjusted OR for MARTA (quetiapine and olanzapine) was not significant.

**Table 4:** Propensity score-adjusted odds ratios of drugs categorized into three groups (*N* = 267)

	Odds ratio (95% CI)	P value
TYP (*N* = 137)	1.00 (reference)	
MARTA (*N* = 74)	1.40 (0.66 to 2.96)	0.38
SDA (*N* = 56)	1.12 (0.46 to 2.75)	0.81

*CI* confidence interval, *TYP* typical antipsychotics (chlorpromazine and haloperidol), *MARTA* multi-acting receptor-targeted antipsychotics (olanzapine and quetiapine), *SDA* serotonin dopamine antagonists (perospirone and risperidone)

### Subgroup analysis for patients who could receive oral drug administration

Supplementary Table 1 provides the number of patients receiving each drug stratified by oral intake availability. The non-oral group primarily received haloperidol, attributable to the limited options of parenteral antipsychotics in Japan [25]. Given the small overlap in the box plots for haloperidol, as shown in Fig. 1, we conducted a supplementary analysis on the orally available subgroup to complement the primary analysis.

Supplementary Table 2 presents the adjusted ORs for each drug in the orally available subgroup. Similar to the primary results, olanzapine exhibited a relatively large OR, though insignificant. Supplementary Table 3 shows the OR of the three categories of antipsychotics. The adjusted OR for MARTA was not statistically significant.

## Discussion

In this secondary analysis of a multicenter prospective observational study of delirium in patients with advanced cancer, olanzapine showed a significant OR in reducing the RASS-PAL scores on day 3 among patients with agitated delirium, as indicated in the logistic regression model with multiple propensity scores as a covariate. The OR for MARTA, which included olanzapine and quetiapine, was not statistically significant.

This study showed an association between olanzapine and a reduction in RASS-PAL scores on day 3. In contrast, our previous study found that quetiapine significantly reduced DRS-R98 scores in the same Phase-R dataset [7]. Another prior study using the same dataset suggested that drug selection has no impact on predicting the improvement in DRS-R98 scores [8]. These findings suggest that the management strategy for delirium may depend on the indicator used to assess the outcome, namely a comprehensive evaluation of delirium symptoms (i.e., DRS-R98) or a specific focus on agitation (i.e., RASS). While most randomized controlled trials (RCTs) on delirium symptom management have used the DRS-R98 as a primary endpoint [10, 26, 27], recent studies have used the RASS as an outcome, such as RCTs examining the efficacy of pharmacotherapy [15, 16] and research on treatment algorithms for agitated delirium [28]. Our study further highlights the importance of developing evidence-based approaches to managing agitated delirium in patients with advanced cancer.

A subgroup analysis was conducted focusing on patients capable of receiving oral pharmacotherapy, the need for which was suggested by the small overlap of multiple propensity score distributions. In addition to the results of the main analysis, this subgroup analysis also showed a relatively large OR for olanzapine. Notably, a historic RCT highlighted the cognitive impairment associated with the anticholinergic effects of antipsychotics used for delirium [29]. Olanzapine possesses a moderate level of anticholinergic side effects among antipsychotics [30], which might render it less ideal for selection. Nonetheless, this study in real-world settings suggests that olanzapine might be preferred for reducing agitation in patients with advanced cancer.

The effectiveness of olanzapine for delirium in palliative care settings has been investigated but remains controversial [31–33]. Frequent sedation as a side effect of olanzapine has also been noted [31]. Balancing sedation and communication capacity is vital for patients with a limited prognosis [20], and personalization based on sedation preference in the patients’ surroundings is also critical [21]. Moreover, the predominant use of olanzapine in a specific center might have influenced the results through certain institutional practices. Furthermore, the effect size was not significant when the drugs were combined and categorized as MARTA. These may require a cautious interpretation of the findings on olanzapine.

This study had several limitations. First, wide confidence intervals in the ORs were observed, presumably because of the small sample size of patients receiving each drug. Second, we were unable to examine the distinct effects of perospirone and trazodone, and only five antipsychotics were included in the main analysis. Third, although the analysis was adjusted for settings by adding a dichotomous variable indicating palliative care or psycho-oncology to the propensity score calculation, unmeasured confounders related to facilities might have influenced the results. Such confounds may include clinicians’ preferences for delirium management, such as prioritizing sedation over cognitive improvement. Fourth, we were unable to include several factors that may affect the course of delirium, such as polypharmacy [34]. Finally, the analysis did not thoroughly consider the trade-off between efficacy and safety, exemplified by a previous study revealing that quetiapine possesses both high efficacy of treatment and low risk of QTc prolongation [35]. Future studies should conduct a further comprehensive evaluation of the risk–benefit profile. Despite these limitations, this study constitutes one of the few investigations on agitated delirium in patients with advanced cancer in real-world settings.

In summary, our findings suggest that the management strategy for delirium in patients with advanced cancer depends on outcome measurement, namely, comprehensive delirium symptoms or a sole focus on agitation. In patients with agitated delirium, olanzapine may effectively reduce agitation. However, the study limitations, such as the small sample size and potential confounders from the facilities, underscore the need for further research to validate our findings.

### Supplementary Information

Below is the link to the electronic supplementary material.

Supplementary file1 (PDF 168 KB)

## Data Availability

The datasets are available from the corresponding author upon reasonable request.

## Code availability

The source codes are available from the corresponding author upon reasonable request.
