# Supplementary material for: Effectiveness of antipsychotics for managing agitated delirium in patients with advanced cancer: a secondary analysis of a multicenter prospective observational study in Japan (Phase-R)
Source: Support Care Cancer. 2024 Feb 8;32(3):147. doi: 10.1007/s00520-024-08352-2 (PMC10850172; doi:10.1007/s00520-024-08352-2)

Supplementary Table 1. Number of drugs administered to groups by oral intake availability

	Oral intake available	Oral intake unavailable
Chlorpromazine	9 (5.5)	21 (19.8)
Haloperidol	32 (19.4)	75 (70.8)
Olanzapine	20 (12.1)	9 (8.5)
Perospirone	7 (4.2)	0 (0.0)
Quetiapine	44 (26.7)	1 (0.9)
Risperidone	49 (29.7)	0 (0.0)
Trazodone	4 (2.4)	0 (0.0)

Supplementary Table 2. Propensity score-adjusted odds ratios of each drug among patients who can receive drugs orally (N = 154)

	Odds ratio (95% CI)	P value
Haloperidol (N = 32)	1.00 (reference)	
Chlorpromazine (N = 9)	1.99 (0.17 to 26.01)	0.58
Olanzapine (N = 20)	3.60 (0.85 to 16.60)	0.087
Quetiapine (N = 44)	0.69 (0.20 to 2.39)	0.56
Risperidone (N = 49)	0.92 (0.31 to 2.81)	0.88

CI, confidence interval

Supplementary Table 3. Propensity score-adjusted odds ratios of drugs categorized into three groups among patients who can receive drugs orally (N = 161)

	Odds ratio (95% CI)	P value
TYP (N = 41)	1.00 (reference)	
MARTA (N = 64)	1.10 (0.43 to 2.86)	0.84
SDA (N = 56)	0.96 (0.36 to 2.57)	0.94

CI, confidence interval; TYP, typical antipsychotics (chlorpromazine and haloperidol); MARTA, multi-acting receptor-targeted antipsychotics (olanzapine and quetiapine); SDA, serotonin dopamine antagonists (perospirone and risperidone)

Supplementary Figure 1. Number of facilities according to the number of patients receiving each drug. A: Chlorpromazine, B: Haloperidol, C: Olanzapine, D: Quetiapine, and E: Risperidone.

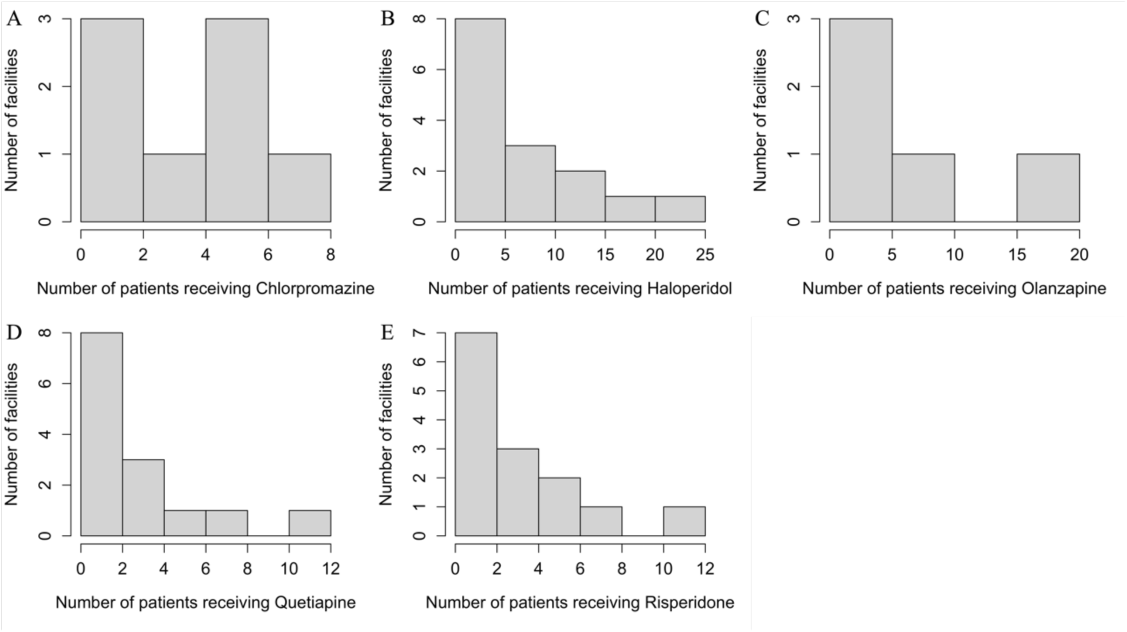

Supplement: Supplementary file 1 — Supplementary file1 (PDF 168 KB) [file 520_2024_8352_MOESM1_ESM.pdf]
